# Clarified açaí (*Euterpe oleracea* Martius) exerts protective effects against methylmercury toxicity in salivary glands and total saliva

**DOI:** 10.1007/s10534-026-00793-y

**Published:** 2026-02-20

**Authors:** Vinicius Ruan Neves dos Santos, Leonardo Oliveira Bittencourt, Hadassa Helez Neves Ferreira, Daiane Claydes Baia-da-Silva, Paulo Fernando Santos Mendes, Luis Eduardo de Oliveira Teixeira, Antonio Hernandes Chaves-Neto, Bruno Santana Carneiro, Diomar Cavalcante Oliveira, Iracina Maura de Jesus, Renata Duarte de Souza-Rodrigues, Herve Rogez, Rafael Rodrigues Lima

**Affiliations:** 1 Laboratory of Functional and Structural Biology, Institute of Biological Sciences, Federal University of Pará, Augusto Corrêa Street, GuamáBelém, Pará 66075-110 Brazil; 2 Faculty of Food Engineering and Center for Valorization of Bioactive Compounds from the Amazon, Federal University of Pará, Belém, Pará Brazil; 3 Department of Basic Sciences, School of Dentistry, São Paulo State University, Araçatuba, Brazil; 4 Environment Section, Evandro Chagas Institute, SAAMB/IEC, Ananindeua, Pará Brazil

**Keywords:** Mercury, Anthocyanin, Phenolic compound, Oral health, Amazon biodiversity

## Abstract

Methylmercury (MeHg) contamination can cause damage to the salivary glands, which is associated with oxidative stress and glandular dysfunction. Açaí (*Euterpe oleracea* Martius), a fruit rich in antioxidants, emerges as a natural alternative to mitigate the toxic effects of MeHg. This study aimed to evaluate whether clarified açaí juice exerts a protective effect on the major salivary glands of rats intoxicated with MeHg. Wistar rats were allocated into four groups: control, MeHg-exposed (0.04 mg/kg/day), açaí-supplemented (0.01 L/kg/day), and MeHg-exposed with açaí supplementation. The compounds were administered by orogastric gavage for 60 days. Subsequently, total saliva was collected to determine antioxidant capacity, lipid peroxidation, and amylase activity, while submandibular and parotid glands were analyzed to total mercury (Hg) levels, antioxidant capacity, and lipid peroxidation. Our results showed that açaí supplementation did not reduce Hg accumulation in the salivary glands. However, MeHg exposure significantly decreased antioxidant capacity in both glands, whereas açaí supplementation mitigated this reduction, maintaining values comparable to the control group. Lipid peroxidation was elevated in the MeHg group in both glands, but this alteration was attenuated by açaí. In saliva, MeHg exposure lowered antioxidant capacity and elevated lipid peroxidation levels, both of which were attenuated by açaí supplementation. Moreover, MeHg altered salivary protein concentration and reduced amylase activity, while açaí supplementation counteracted these effects. These results demonstrate that açaí’s antioxidant constituents confer protective effects against MeHg-induced oxidative damage and functional impairment in salivary glands and saliva, underscoring its potential as a natural protective agent against Hg toxicity.

## Introduction

Human exposure to mercury (Hg) is recognized as a global public health issue, affecting various regions around the world (WHO 2021). South America is the second-largest emitter of anthropogenic Hg into the atmosphere, with the Amazon region accounting for more than 78.5% of the continent's Hg emissions (UNEP 2019; Galvis 2020). Industrial activities and artisanal gold mining remain the primary anthropogenic sources that increase Hg bioavailability in the biosphere, contributing to the biomagnification process in living organisms (Yoshimura et al. 2021; Nascimento et al. 2021a). As a result, Hg ranks third among the most significant chemical threats to human health and the environment due to its numerous adverse effects on biological systems and ecosystems (WHO 2021; ASTDR 2023). Consequently, Hg contamination in communities residing in the Amazon biome, particularly in the form of methylmercury (MeHg), is a major concern, once the exposure to the metal occurs mainly through oral ingestion of a diet rich in fish, as well as through inhalation of Hg vapors during occupational exposure in artisanal gold mining, leading to serious health risks (Yoshimura et al. 2021).

Among the health impacts associated with human exposure to Hg and its organic and inorganic species, particularly MeHg, evidence indicates that this toxic metal exerts highly heterogeneous effects depending on its chemical species, dose, exposure duration, and cellular context (Wu et al. 2024). At the cellular level, Hg disrupts redox homeostasis through its strong affinity for sulfhydryl groups, leading to depletion of antioxidant defenses, mitochondrial dysfunction, calcium imbalance, and activation of pro-inflammatory and apoptotic pathways (Farina et al. 2011; Perrone et al. 2025). As a consequence, these redox-mediated disturbances compromise essential cellular processes and have been consistently reported in multiple cell types, including epithelial (Rodríguez-Viso et al. 2022), neuronal (Puty et al. 2023), endothelial (Perrone et al. 2024), and glandular cells (Nogueira et al. 2021).

Within this framework, increasing evidence from in vivo studies has demonstrated that salivary glands are particularly susceptible to Hg-induced toxicity under conditions of prolonged exposure. In recent years, our research group has highlighted the vulnerability of these glands to Hg and MeHg exposure, showing structural, biochemical, and functional impairments in experimental models (Aragão et al. 2022; Nascimento et al. 2021a; Farias-Júnior et al. 2017; Bittencourt et al. 2017). The salivary glands, classified as parotid, submandibular, and sublingual glands, play essential roles in oral physiology, and the major glands account for approximately 92–95% of total saliva production (Varga 2012).

Saliva is a complex biological fluid that is fundamental to oral and systemic homeostasis. It contributes to lubrication of the oral mucosa, facilitation of mastication and swallowing, initial carbohydrate digestion through enzymes such as amylase, buffering and pH regulation, antimicrobial defense, maintenance of oral microbiota balance, and protection of dental tissues against demineralizing agents (Peyrot Des Gachons and Breslin 2016; de Paula et al. 2017; Dawes et al. 2015; Boehlke et al. 2015). Preclinical evidence indicates that Hg intoxication disrupts these physiological functions by impairing salivary gland activity, resulting in alterations in salivary flow, protein composition, and enzymatic activity, including changes in amylase levels (Nascimento et al. 2021b).

Disruption of these salivary functions may compromise oral and digestive processes, favoring conditions such as xerostomia, microbiota dysbiosis, impaired initial carbohydrate digestion, and increased susceptibility to dental caries (Dawes et al. 2015; Boehlke et al. 2015). Ultimately, persistent salivary gland dysfunction has been associated with reduced quality of life, as alterations in saliva quantity and composition negatively affect speech, mastication, taste perception, swallowing, and sleep, while increasing the risk of oral infections and mucosal lesions (Kapourani et al. 2022; Müller et al. 2023; Alnaeem et al. 2025).

The search for preventive and therapeutic strategies against diseases caused by MeHg has seen significant effort in the literature (Moniruzzaman et al. 2021). Given this fact, açaí (*Euterpe oleracea* Mart.) has stood out in the world not only for its high nutritional value, but also for its high antioxidant capacity and its potential for the development of new products with therapeutical effects (Lichtenthäler et al. 2005; Rodrigues et al. 2006; Pacheco-Palencia et al. 2007; Souza-Monteiro et al. 2019; Dos Santos et al. 2022). Among the bioactive polyphenols present in açaí, anthocyanins stand out for their ability to inhibit the action of free radicals responsible for oxidative stress - an important mechanism of damage related to exposure to MeHg - in addition to affecting the anti-inflammatory response and reducing tissue damage (del Pozo-Insfran et al. 2004; El-Shinnawi and Soory 2015; Dos Santos et al. 2022). In the study by Crespo-Lopez et al. (2019), clarified açaí is investigated as a possible protector of the central nervous system against damage caused by exposure to MeHg, and the results point to less damage in animals that received açaí supplementation. Thus, the choice of açaí as a functional food for this research goes beyond its known phytochemical properties, but is also based on previous studies, its vast availability in the soils of the Amazon region and its high consumption and exportation to several others countries (Rogez 2000; Bichara and Rogez 2011).

Therefore, considering the biotechnological potential of clarified açaí due to its high content of antioxidant molecules and previous studies that demonstrated the therapeutic effects of açaí, this study aimed to investigate whether supplementation with clarified açaí, concomitant with chronic intoxication by MeHg exerts protective effects on the major salivary gland. It was hypothesized that clarified açaí, when administered systemically and for a long period, protects the parotid and submandibular glands against biochemical damage, evaluating lipid peroxidation (LPO) and antioxidant capacity against peroxyl radicals (ACAP) levels, and functional damage by evaluating amylase activity triggered by MeHg.

## Materials and methods

### Ethical aspects

The experimental protocol of this research was submitted and approved by the Ethics Committee on the Use of Animals of the Federal University of Pará under protocol number 5307280722 A, in line with the Arrive 2.0 guide (Percie Du Sert et al. 2020).

### Clarified açaí (*Euterpe oleracea*)—production and characteristics

**Fig. 1:**
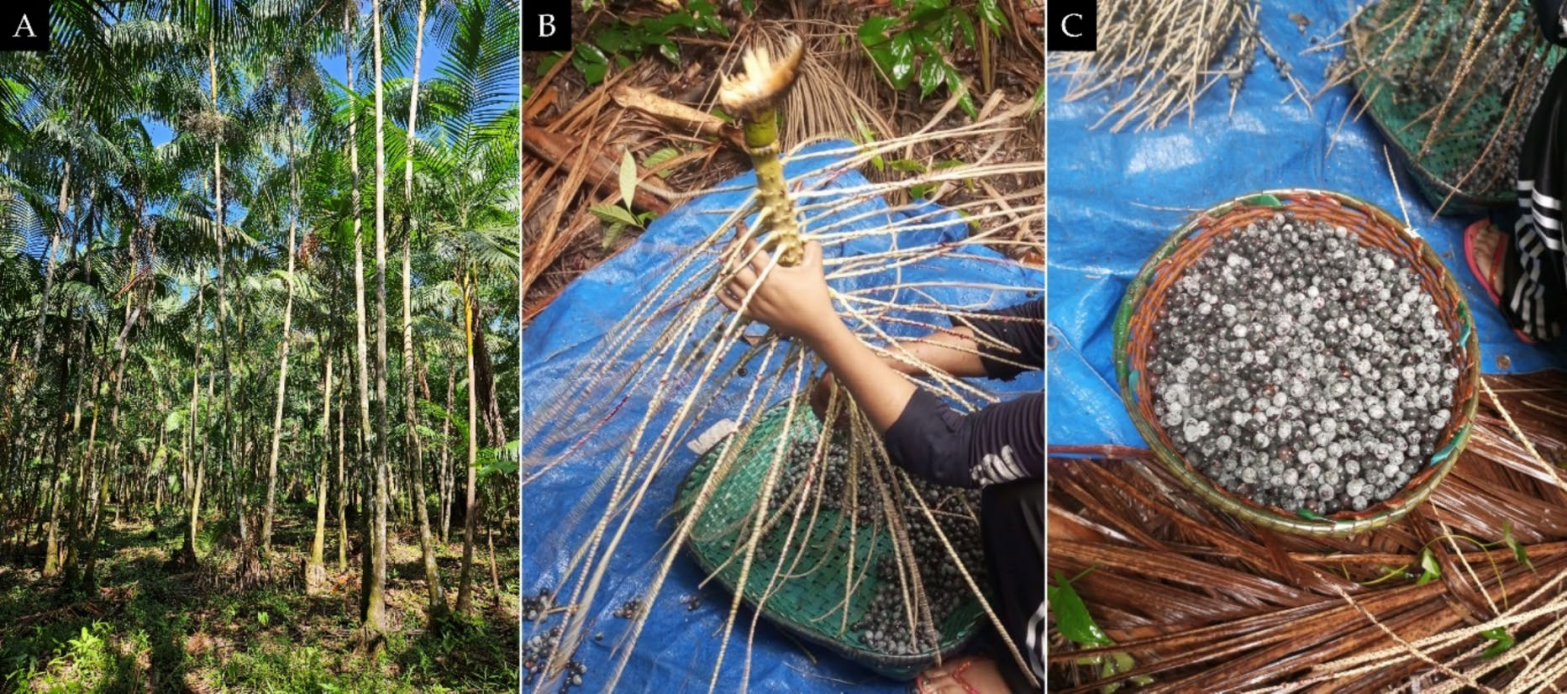
Açaí (*Euterpe oleracea* Martius). In **A**, view of the *Euterpe oleracea* palm plantation where açaí is obtained; In **B**, manual process of removing the açaí drupes from the bunch; in **C**, black açaí, at which stage it is used to prepare the pulp

The clarified juice of *Euterpe oleracea* used in this study was kindly provided by the company Amazon Dreams (Belém, Pará, Brazil). The patented process to produce the extract was previously licensed by Amazon Dreams and Federal University of Pará (PI 8 1003060-3). This process includes microfiltration and centrifugation of açaí pulp to form a juice, prepared with fresh drupes (Fig. 1). In an aliquot of this juice, total polyphenols were quantified by the Folin–Ciocalteu method, a colorimetric assay based on the reducing action of polyphenols in the sample against the Folin-Ciocalteu phenol reagent; gallic acid is used as the calibration standard, and the results are expressed as milligrams of gallic acid equivalents (GAE) per 100 g of fruit (Pompeu et al. 2009; Singleton and Rossi 1965). In addition, the content of major flavonoids was characterized by high-performance liquid chromatography (HPLC) (Snyder et al. 2011). This study is registered on the Brazilian government platform National System for the Management of Genetic Heritage and Associated Traditional Knowledge (SISGEN) under number AAAFF39.

In addition, açaí was evaluated for its Trolox Equivalent Antioxidant Capacity (TEAC) by the ABTS^·^ + and DPPH^·^ methods (Miller et al. 1993; Re et al. 1999; Blois 1958). The ABTS^·^ + radical scavenging assay was carried out following the methodology adapted from Miller et al. (1993) and later modified by Re et al. (1999). The ABTS^·+^ solution (2,2′-Azino-bis (3-ethylbenzothiazoline-6-sulfonic acid) was prepared by mixing 7 mM ABTS^·^+ with 140 mM potassium persulfate (K₂O₈S₂), followed by incubation at room temperature in the absence of light for 16 h. After this period, the solution was diluted using a phosphate-buffered saline solution until reaching an absorbance of 0.700 (± 0.02) at 734 nm. To establish the calibration curve, the synthetic antioxidant Trolox (6-hydroxy-2,5,7,8-tetramethylchromono-2-carboxylic acid) was used as a reference standard. For the antioxidant capacity assessment, 2.97 mL of the ABTS^·+^ solution was transferred to a cuvette, and its initial absorbance at 734 nm was measured using an Nm Kasvi spectrophotometer. Following this step, 0.03 mL of the test sample was added to the cuvette containing the ABTS^·+^ radical. After a 5-min reaction period, a second absorbance reading was recorded to determine the antioxidant activity.

The DPPH assay was conducted to evaluate the ability of açaí to neutralize the 1,1-diphenyl-2-picrylhydrazyl (DPPH^·^) radical, a violet-colored chromophore that, upon reduction, transforms into its hydrogenated form, appearing yellow or colorless. The assay was performed based on the methodology described by Blois (1958). To assess the antioxidant activity, the initial absorbance of a 0.1 mM DPPH^·^ solution (2,2-diphenyl-1-picrylhydrazyl) diluted in ethanol was recorded. Following this, a mixture containing 0.6 mL of DPPH^·^ solution, 0.35 mL of distilled water, and 0.05 mL of the test sample was prepared and incubated in a water bath at 37 °C for 30 min. After incubation, the absorbance readings were taken using an Nm Kasvi spectrophotometer at λ 517 nm. We used Trolox (6-hydroxy-2,5,7,8-tetramethylchromono-2-carboxylic acid), a synthetic antioxidant, to construct the standard curve. The results were expressed in mM, and the values obtained for the samples were compared to the 1 mM Trolox standard.

### Experimental animals

**Fig. 2:**
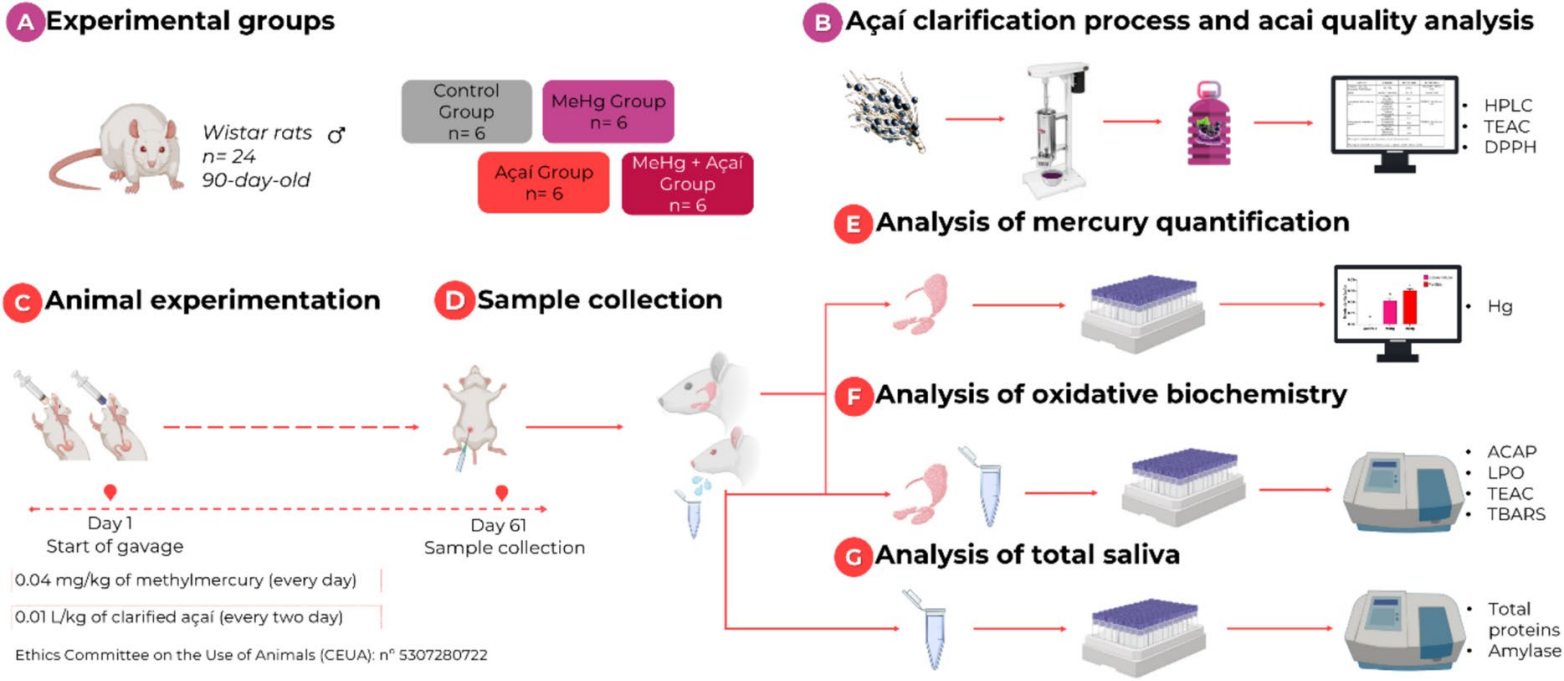
Overview of the experimental protocol. In **A**, division of experimental groups; in **B**, the clarification process before animal experimentation; in **C**, the period of animal experimentation where animals were intoxicated with MeHg (0.04 mg/kg) and supplementation with clarified juice of açaí (0.01 L/kg); in **D**, collection of submandibular and parotid glands and saliva; in **E**, Hg quantification analysis in the salivary glands; in **F** oxidative biochemical evaluation of the salivary glands and total saliva; in **G**, morphometric analysis of the salivary glands and in **H**, analysis of total proteins (TP) in saliva

Male Wistar rats with 90-day-old (n = 24; weight ≅ 180–220 g) were housed in groups of four animals in polypropylene collective cages. The animals were maintained under appropriate temperature conditions of 25 ± 2 °C, with a 12-h light/dark cycle, with food (Presence, Neovia, Brazil) and water ad libittum. During the experimental period, the animals were monitored twice a day for their health status. No complications were observed. The animals were divided by simple randomization into four experimental groups (n = 6 per group): control group, MeHg intoxicated group (MeHg); group only supplemented with clarified açaí (Açaí), and group intoxicated with MeHg and supplemented with clarified açaí (MeHg + Açaí). The experimental design and analysis performed are summarized in Fig. 2.

### MeHg exposure protocol

The animals in the MeHg and MeHg + Açaí groups received, daily, by orogastric gavage, MeHg chloride (CAS No. 115-09-3, Sigma-Aldrich, Saint Louis, MO, USA) solubilized in corn oil at a dosage of 0.04 mg/kg body weight/day of MeHg. The intoxication occurred throughout the experimental period (60 days), following the protocol validated in previous studies by our research group (Bittencourt et al. 2017; de Oliveira Lopes et al. 2021; Farias-Junior et al. 2017; Lima et al. 2018). The volume of oil received in the control group and Açaí group was similar to the volume of the exposed group during the same experimental period (60 days). Animals belonging to the other groups (control and açaí groups) received corn oil in a proportional volume and for the same period.

### Açaí supplementation protocol

The animals in the açaí and MeHg + Açaí groups received clarified açaí by orogastric gavage at doses of 0.01 L/kg body weight for 60 days on alternate days, totaling 30 doses of supplementation. The protocol was adapted from previous studies that demonstrated the antioxidant efficacy of this dose in other injury models without animal losses during experimentation (da Silva et al. 2023; dos Santos et al. 2022; De Moura et al. 2025). Animals belonging to the other groups (control and MeHg groups) received distilled water in a proportional volume for the same period.

### Sample collection and preparation

After the experimental period, the animals were anesthetized with ketamine hydrochloride (90 mg/kg) and xylazine hydrochloride (9 mg/kg). Shortly after the loss of all corneal and paw reflexes, salivation was stimulated by intraperitoneal injection of pilocarpine (1 mg/kg). Saliva was collected in ice-cooled plastic tubes for 10 min after the appearance of the first drop and subsequently stored at − 80 °C for up to 20 days before analysis (Narita et al. 2019; Nascimento et al. 2021b). After collecting the saliva, the animals were euthanized by exsanguination. The submandibular and parotid salivary glands intended for Hg quantification and oxidative stress analysis were collected fresh and immediately frozen in liquid nitrogen and subsequently stored in a -80ºC freezer until analysis is performed and stored in a  − 80 °C freezer. The samples were identified in codes for subsequent blinding of the analyses.

### Hg concentration measurement in the salivary glands

To confirm animal exposure, total Hg was determined in salivary gland samples. Total Hg was quantified by cold vapor atomic absorption spectrometry (CVAA), following the Suzuki and Akagi (2004) protocol, and used a certified material of analytical quality control (DORM-3). For this analysis, 0.25 g of wet sample was subjected to digestion in a solution composed of nitric acid, perchloric acid and sulfuric acid in a 1:1:5 (v:v:v) ratio. This digestion was carried out on an electric hot plate heated to 210 °C for 30 min. After this process, the total volume of the solution was adjusted to 50 mL with ultrapure water and the samples were analyzed. To convert Hg ions (Hg^2^⁺) into elemental Hg (Hg^0^), a chemical reduction with stannous chloride (SnCl_2_) was used. Thus, the vapor generated was automatically directed to the absorption cell after cooling, circulating continuously through a diaphragm system until the cell where the reading occurred. Thus, Hg detection was performed by means of optical absorption at 253.7 nm, with the results obtained from comparison with a standard curve. Measurements of Hg concentration in the samples were expressed μg/g.

### Oxidative stress assessment in salivary glands

The samples were thawed and resuspended in 20 mM Tris–HCl (pH 7.4, at 4 °C), and then homogenized by sonic disaggregation. Then, lysate was centrifuged for 10 min at 3000 rpm and the supernatant was collected and stored at − 80 °C until biochemical analysis. From the crude homogenate of the submandibular and parotid glands and the total saliva collected, the protein concentration in these samples was determined using the Bradford method (1976) and the results were expressed as g/dL.

To determine the concentration of total proteins in salivary glands, the method proposed by Bradford (1976). This technique is based on the interaction of proteins with the Coomassie Brilliant Blue G-250 dye, forming a blue colored complex with maximum absorbance at λ 595 nm.

The antioxidant capacity against peroxyl radicals (ACAP) of gland was performed the fluorometric method previously described by Amado et al. (2009) on a fluorimeter (Victor X3, Perkin Elmer). The results obtained were expressed as the inverse of the relative area and subsequently converted to a percentage of the control. Subsequently, the lipid peroxidation (LPO) was assessed by determining the malondialdehyde levels in salivary glands according to the methodology proposed by Esterbauer and Cheeseman (1990). The analyses were carried out using a multimode reader (Victor X3, Perkin Elmer). The results were plotted as a percentage of the control and are represented in nmol/µg after correction by protein concentration.

### Assessment of oxidative stress in the saliva

In addition to the analyses of the glands, we sought to investigate whether changes in the salivary glands would affect the oxidative biochemical balance of saliva. To this end, the TEAC was first determined, as previously described in the subsection “Clarified açaí (*Euterpe oleracea*)—production and characteristics” of this session. The analyses were carried out in a spectrophotometer (K37-VIS, Kasvi), and the results were expressed in μM/g of protein, and then converted to a percentage of the control group. Then, the LPO was measured by the protocol of Kohn and Livesedge (1944) and adapted by Percário et al. (1994), which consists of measuring the thiobarbituric acid reactive species (TBARS). Results were expressed in nM/g of protein and then converted to a percentage of control.

### Assessment of salivary amylase activity and determination of protein concentration

When evaluating the possible functional effects, we analyzed the activity of amylase, an important enzyme produced by the major salivary glands and closely involved in the digestion of carbohydrates. Amylase activity was determined according to a modified method from Caraway (1959) by colorimetric detection (K003 BIOCLIN kit, Quibasa, Belo Horizonte, Minas Gerais, Brazil). Absorbance measurements were carried out by spectrophotometry (K37-VIS, Kasvi) at 660 nm and amylase activity was expressed as Units/g of protein.

### Statistical analyses

To perform the sample calculation, the program was used G*Power software (Statistical Power Analyzes 3.1.9.2). The mean and standard deviation values of the lipid peroxidation evaluation from the study by Farias-Júnior et al. (2017) were used to determine the effect size. In addition, an alpha value of 0.01, a power of 95% and a loss of 20% were considered, resulting in a size of 6 animals per group. After carrying out all tests, the data were tabulated in the GraphPad Prism 8.0.2 software. The data were analyzed using the Shapiro-Wilk normality test, considering *p* > 0.05. Data with normal distribution were submitted to the one-way ANOVA test followed by Tukey’s post-test, with all analyses considering the statistical significance value *p* < 0.05. The oxidative biochemistry results were normalized by protein concentration and expressed as a percentage of the control with mean ± standard error of the mean, while the other results were expressed as mean ± standard error of the mean with their respective measurement units. Pearson's correlation test assessed the correlation between the obtained parameter data. In addition, Multivariate Analysis of Variance (MANOVA) was performed to test the validity of the hypothesis that the means of the dependent variables were different between the experimental groups. To assess the significance of the differences between the groups in this study, the Wilks lambda test was applied, and a *p* value < 0.05 was considered statistically significant (Tabachnick and Fidell 2013). The jamovi software (version 2.6.13.) was used to evaluate the data.

## Results

### Analysis of clarified açaí juice composition

The total content of phenolic compounds was 273.82 mg gallic acid eq./100 g. The main phenolic compounds of the clarified açaí sample, using HPLC–DAD methods, were identified and quantified as cyanidin-3-rutinoside (29.58 mg/100 mL), homoorientin (2.04 mg/100 mL), orientin (1.27 mg/100 mL), cyanidin-3-glucoside (8.96 mg/100 mL) and taxifolin deoxyhexose (0.00 mg/100 mL). The antioxidant capacity of the sample, evaluated by the ABTS^·^ + and DPPH^·^ methods were 0.201 ± 0.004 mM and 0.721 ± 0.029 mM, respectively (expressed as mean ± standard error).

### The supplementation with clarified açaí did not after Hg accumulation in both salivary glands

As described in Table 1, total Hg concentrations were significantly increased in the salivary glands of intoxicated groups compared to non-intoxicated controls. In addition, no statistically significant differences were observed between the MeHg + açaí and MeHg groups, indicating that açaí supplementation did not alter mercury levels in exposed animals in either the parotid or submandibular glands.

**Table 1:** Total Hg (THg) concentration in submandibular and parotid gland

Group	THg concentration (µg/g)	
	Submandibular	Parotid
Control	0.002 ± 0.0009^a^	< L.Q.^a^
Açaí	< L.Q.^a^	< L.Q.^a^
MeHg	0.469 ± 0.050^b^	0.447 ± 0.023^b^
MeHg + Açaí	0.592 ± 0.051^b^	0.562 ± 0.053^b^

*Note* Each gland was analyzed separately

Values expressed as mean ± standard error of mean (S.E.M.), 1-way ANOVA test with Tukey post-test (n = 6)

L.Q..: Limit of quantification equivalent to 0.118 ng/g (0.000118 µg/g)

^a,b^Different superscript letters indicate statistical difference (*p* < 0.05)

### Clarified açaí supplementation protected the salivary glands against redox imbalance triggered by long-term exposure to MeHg

**Fig. 3:**
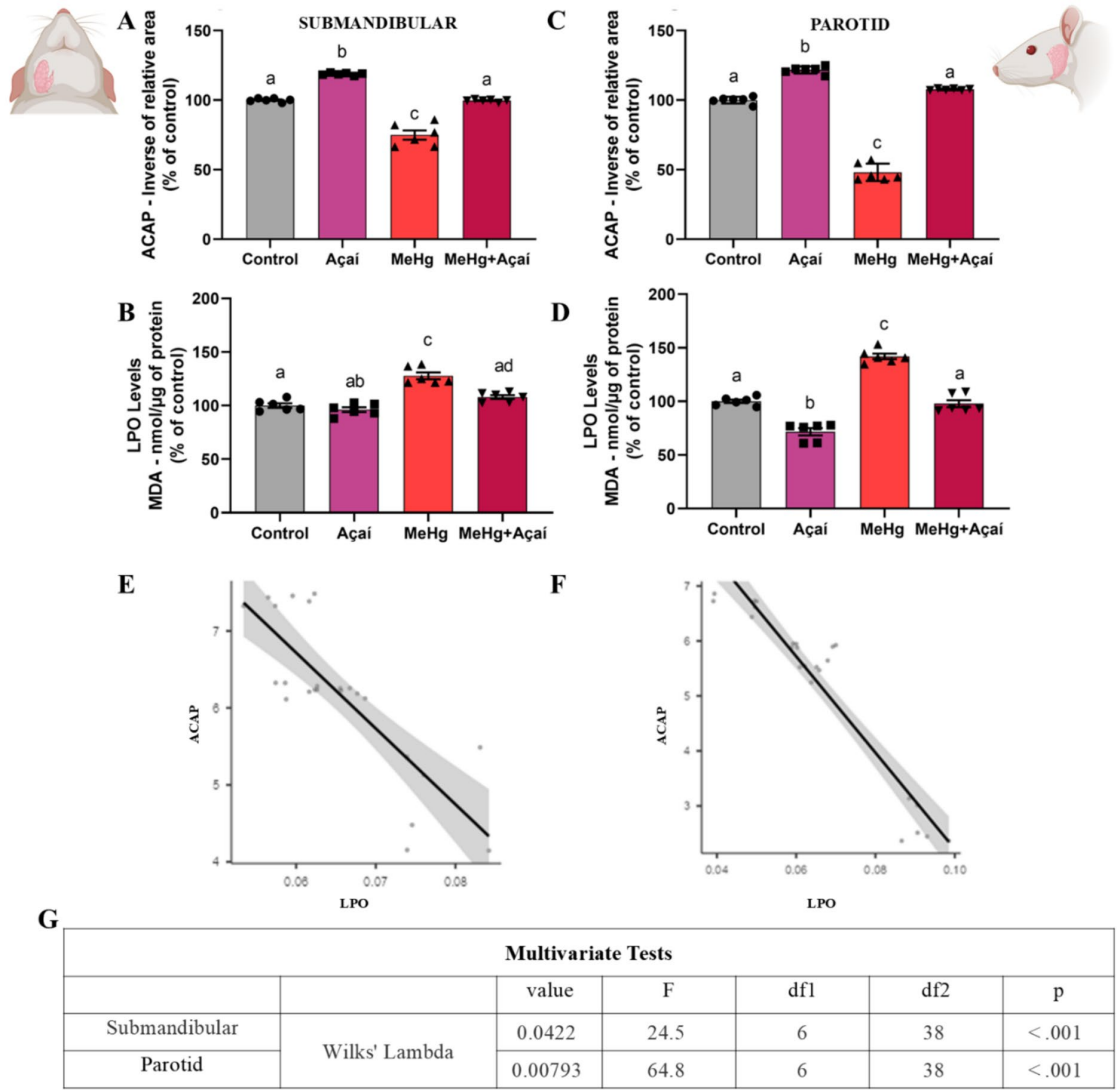
Effects of long-term exposure to MeHg (0.04 mg/kg/day) and supplementation with clarified açaí (0.01 L/kg/day) on the oxidative biochemistry of the submandibular and parotid glands. In **A**, Antioxidant capacity against peroxyl radicals (ACAP) of the submandibular gland (% of control); In **B**, lipid peroxidation (LPO) of the submandibular gland (% of control); in **C**, ACAP of the parotid gland (% of control); In **D**, LPO of the parotid gland (% of control). Quantitative results are expressed as mean ± standard error of the mean, 1-way ANOVA test with Tukey post-test (n = 6). Different letters mean statistical difference (*p* < 0.05). In **E**, ACAP-LPO correlation graph of the submandibular; In **F**, ACAP-LPO correlation graph of the parotid; In **G**, Table with the results of the multivariate Wilks Lambda test in the glands. In the upper right corner, an illustrative image depicts the anatomical location of the submandibular gland, and in the upper left corner, another image illustrates the location of the parotid gland

The ACAP of submandibular gland in MeHg intoxicated group was reduced in approximately 23.25 ± 3.35% in comparison to control group (100.0 ± 0.520%; *p* < 0. 0001). When analyzing the effects of açaí supplementation, the ACAP was unaltered in comparison to control (*p* = 0.9985). In addition, the group supplemented only with clarified açaí (118.5 ± 0.376%) showed higher antioxidant capacity values than the control group (*p* < 0.0001) (Figure 3A).

The LPO in submandibular gland in chronically-exposed group showed an increment of approximately 27.7 ± 3.188% when compared to the control group (100.0 ± 2.053%; *p* < 0.0001). On the other hand, the MeHg + Açaí had lower LPO levels (MeHg + Açaí group; 108.0 ± 1.758%) when compared to the MeHg alone (*p* < 0.0001) and statistically equivalent to control (*p* = 0.1151). Furthermore, no statistical difference was observed between the control and açaí groups (96.20 ± 2.262%; *p* = 0.6750) (Figure 3B).

The ACAP of parotid gland in MeHg-exposed group decreased in approximately 51.95 ± 2.554% in comparison to control (100.0 ± 0.996%; *p* < 0.0001) and was maintained unaltered when the animals were supplemented with açaí despite the intoxication (107.7 ± 0.241%; *p* = 0.0074). Furthermore, it was noticed an increase in ACAP values of approximately 21.6 ± 1.060 % when compared to the control group (*p* < 0.0001) (Figure 3C).

There was a significant increase in LPO in chronically-exposed group (142.0 ± 2.628%) when compared to the control group (100.0 ± 1.617%; *p* < 0.0001). The MeHg + açaí group (97.85 ± 3.309%) showed equivalent levels in comparison to control group (*p* = 0.9485). The açaí group (71.64 ± 3.362%) showed a significant reduction in LPO values when compared even to the control group (*p* < 0.0001) (Figure 3D).

**Fig. 4:**
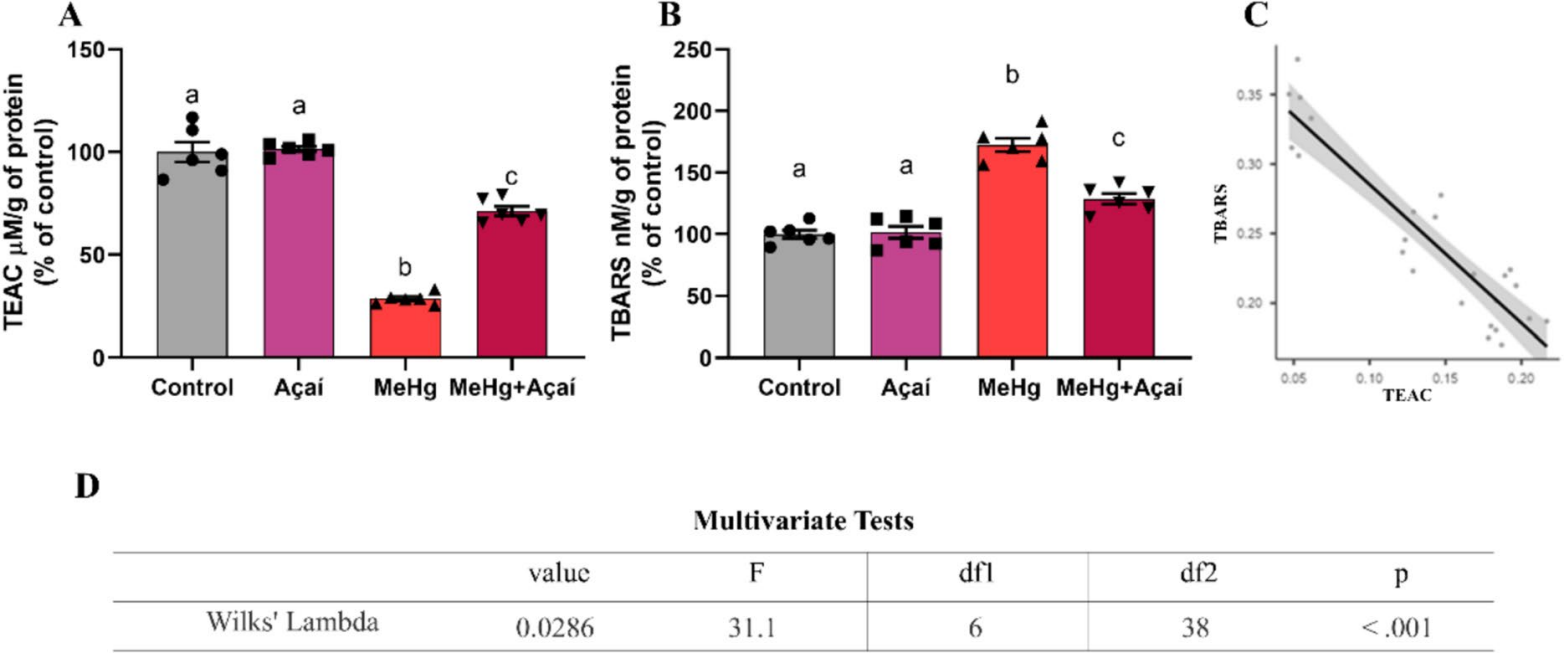
Effects of chronic exposure to MeHg (0.04 mg/kg/day) and supplementation with clarified açaí (0.01 L/kg/day) on the oxidative biochemistry of total saliva. In **A**, Determination of total antioxidant capacity equivalent to Trolox (TEAC; % of control); In **B**, thiobarbituric acid reactive species (TBARS; % of control). Quantitative results are expressed as mean ± standard error of the mean, 1-way ANOVA test with Tukey post-test (n = 6). Different letters mean statistical difference (*p* < 0.05). In **C** TBARS-TEAC correlation graph of saliva; In **D**, Table with the results of the multivariate Wilks Lambda test in the glands

The Person’s correlation test and MANOVA in the biochemistry of the salivary glands showed a negative relationship between ACAP and LPO in submandibular (r: − 0.950, *p* < 0.001) and parotid (r: − 0.822, *p* < 0.001) (Figure 3F). Furthermore, the Wilks' Lambda test indicated a significant multivariate difference between the groups evaluated in both glands (Figure 4G).

### The supplementation with clarified açaí minimized oxidative biochemical imbalances in saliva caused by chronic exposure to MeHg

The antioxidant capacity of saliva in the group exposed to MeHg (28.50 ± 1.124%) was lower in comparison to the control group (100.0 ± 4.750%; *p* < 0.0001). The supplementation with açaí attenuated the TEAC reduction (71.23 ± 2.312%) in comparison to MeHg-exposed (28.50 ± 1.124; *p* < 0.0001). No statistical difference in TEAC levels between the Açaí group (101.4 ± 1.333%) and the control group (*p* = 0.9848) (Fig. 4A). Regarding the LPO in saliva, MeHg + Açaí group (135.0 ± 3.044%) showed a significant reduction compared to the MeHg group (184.0 ± 5.253%; *p* < 0.0001). Both Açaí (108.3 ± 5.102) and control (100.0 ± 2.451%; *p* = 0.5017) groups remained without statistical difference in the evaluation of this parameter (Fig. 4B).

Person's correlation test and MANOVA in whole saliva biochemistry identified a negative relationship between TEAC and LPO, described as the TBARS levels (r: − 0.919, *p* < 0.001) (Fig. 4C). Furthermore, Wilks' Lambda test also indicated a significant multivariate difference between the evaluated groups (Fig. 4D).

### Chronic exposure to MeHg modulated protein concentration and amylase enzyme activity, while supplementation with clarified açaí was able to mitigate this change

**Fig. 5:**
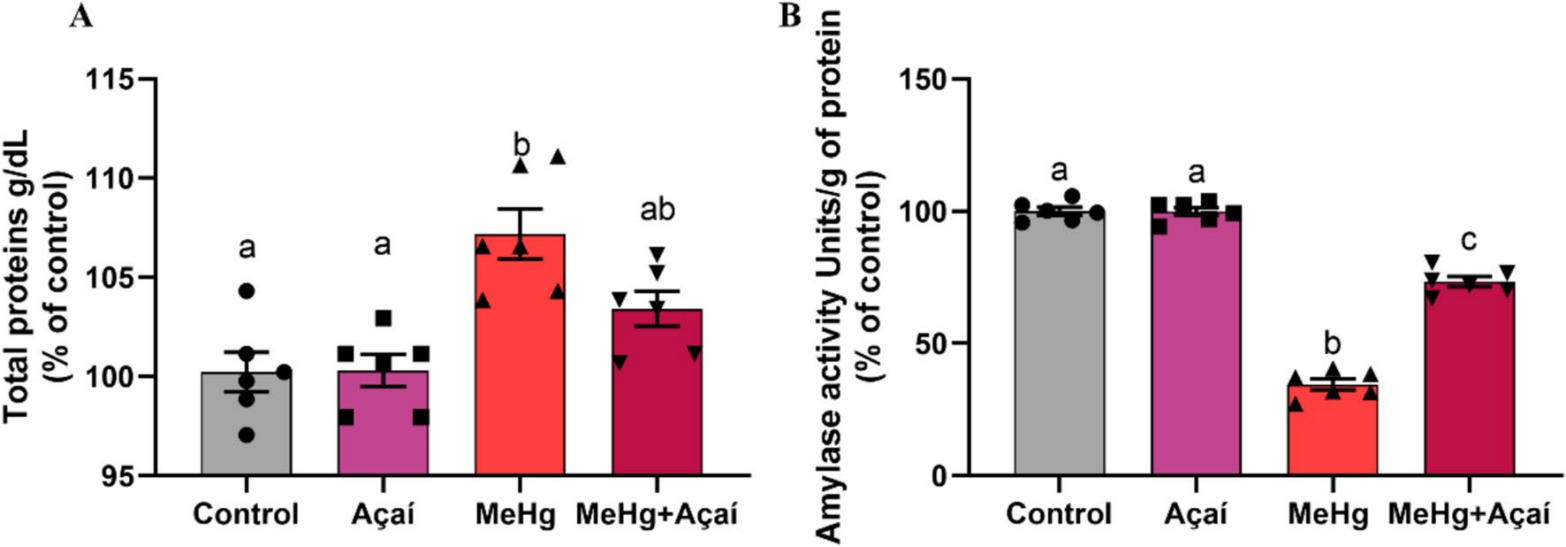
Effects of chronic exposure to MeHg (0.04 mg/kg/day) and supplementation with clarified açaí (0.01 L/g/day) on total protein and salivary amylase values in saliva. In **A**, the quantification of total proteins presents in saliva (g/dL) and, in **B**, the quantification of salivary amylase (% of control). Quantitative results are expressed as mean ± standard error of the mean, 1-way ANOVA test with Tukey post-test (n = 6). Different letters mean statistical difference (*p* < 0.05)

In saliva, the MeHg group (107.2 ± 1.259%) presented higher concentrations of total proteins compared to the control group (100.2 ± 0.993%; *p* = 0.0005) and the açaí group (100.3 ± 0.806%; *p* = 0.0005), while the MeHg + Açaí group (103.4 ± 0.884%) did not express a significant difference in relation to the control (100.2 ± 0.993%; *p* = 0.1457) (Fig. 5A). In addition, the amylase activity in total saliva from the MeHg-exposed group (36.78 ± 2.227%) was reduced in relation to the control (100.0 ± 0.915%; *p* < 0.0001). On the other hand, the group with the MeHg + Açaí combination (76.98 ± 1.998%) showed higher amylase activity values than the MeHg group (36.78 ± 2.227%; *p* < 0.0001). No statistical difference in amylase concentration was observed when comparing the Açaí (100.2 ± 0.822%) and control group (*p* = 0.9996) (Fig. 5B).

## Discussion

This original study investigated the protective effects of clarified açaí juice against MeHg toxicity, with a specific focus on oxidative biochemistry and salivary gland function. Our findings demonstrated that clarified açaí supplementation did not alter Hg bioaccumulation in the salivary glands; however, it significantly enhanced the antioxidant capacity of these glands and reduced lipid peroxidation in both salivary tissue and saliva. Moreover, the decrease in amylase activity observed in animals exposed exclusively to MeHg, possibly associated with the oxidative biochemical alterations detected in the salivary glands and saliva, was attenuated when MeHg exposure was combined with clarified açaí supplementation. Taken together, these findings suggest that the phenolic compounds present in clarified açaí exert a protective effect against MeHg-induced oxidative and functional biochemical damage in the salivary glands. Overall, this evidence indicates that the beneficial effects of clarified açaí are primarily attributable to cytoprotective and antioxidant mechanisms rather than to a reduction in Hg accumulation.

Although other mechanisms of damage to cellular structures are associated with Hg poisoning, such as altered calcium influx and DNA damage, oxidative stress stands out as one of the main mechanisms of damage (Kang et al. 2024; Baia-da-Silva et al. 2024). Oxidative stress conditions in rat salivary glands have already been reported in previous studies by our research group (Fagundes et al. 2016; Bittencourt et al. 2017; Farias-Júnior et al. 2017; Lima et al. 2018; Aragão et al. 2022), whose changes showed that Hg was able to reduce the total antioxidant capacity of the glands and increase the levels of MDA, one of the main pro-oxidant indicators, inducing biochemical-oxidative imbalance. Research suggests that the biochemical and oxidative damage resulting from MeHg poisoning may be associated with its electrophilic properties, which favor interaction with low molecular weight nucleophilic groups, such as thiols and selenols. It is assumed that MeHg may act by interacting with glutathione peptides (GSH), the main low molecular weight thiol found in mammals, interrupting the activity of proteins such as glutathione peroxidase (GSH-Px), thioredoxin (Trx) and thioredoxin reductase (TrxR) (Farina et al. 2011). The interruption of the activity of these proteins would be responsible for the decrease in antioxidant capacity and the increase in the generation of reactive oxygen species (ROS), interrupting the redox balance in cells and, subsequently, leading to lipid peroxidation and damage to cell membranes (Nogueira et al. 2021; Ayala et al. 2014).

Furthermore, among oxidative processes, LPO of membranes can cause structural and permeability changes, leading to the loss of selective ion exchange, the release of organelle contents, and the formation of cytotoxic metabolites such as malondialdehyde (Dardick et al. 1993). In this study, supplementation with clarified açaí reduced the effects of chronic exposure to Mehg, while maintaining LPO levels and antioxidant capacity statistically similar to the control, suggesting that the phenolic compounds present in clarified açaí can reestablish the redox balance in the salivary glands and minimize the damage caused by lipid peroxidation products.

The mechanisms polyphenols present in açaí act to mitigate the effects of Hg exposure is not yet well elucidated in the literature, especially regarding their effects on salivary glands. The phenolic compounds abundantly found in clarified açaí can modulate oxidative stress both directly, by neutralizing free radicals and reactive oxygen and nitrogen species, and indirectly, by modulating endogenous antioxidant systems. Phenolic compounds have hydroxyl groups attached to their aromatic rings, enabling direct action on free radicals, giving one or two H• to stabilize them and prevent oxidative activities. Additionally, chemical intermediates capable of neutralizing reactive species are formed, such as phenolic compounds oxidized by peroxidases, which generate phenoxide radicals that can act as antioxidants (Anggraini et al. 2019; Joaquín-Cruz et al. 2015). The anthocyanins, which in the case of clarified açaí, the cyanidin-3-rutinoside and cyanidin-3-glucoside are the most abundant, exert their antioxidant mechanisms through their conjugated electronic structure and the presence of functional groups, such as hydroxyls. These mechanisms include hydrogen transfer to peroxyl, hydroxyl, and superoxide radicals (Liu et al. 2024), electron transfer for neutralization via resonance, a characteristic of cyclic structures (Olivas-Aguirre et al. 2016), and ion sequestration, acting as an electron acceptor for transition metals such as iron and copper through the Fenton reaction (Zhang et al. 2020; Xie et al. 2018).

Other pathways are related to the increased expression of endogenous antioxidant factors, such as the glutathione system, and the reduction of inflammatory pathways associated with the triggering of oxidative stress mediated by the expression of NF-κB, AMP-activated protein kinase (AMPK), and MAPK. These pathways regulate the expression of pro-inflammatory markers, nitric oxide, cyclooxygenase-2, prostaglandins, and inflammatory cytokines (Jung et al. 2014; Tan et al. 2019). The presence of Hg is associated with the activation of deleterious mechanisms, such as oxidative stress, which can compromise cellular structure and function. Our results, obtained through salivary analysis, showed an increase in LPO, concomitant with a reduction in TEAC in saliva. Furthermore, Pearson's correlation and MANOVA analysis reinforced these findings, demonstrating that elevated LPO levels are inversely related to total antioxidant capacity, characterizing a state of oxidative biochemical imbalance in saliva (Jurczuk et al. 2007; Halliwell and Whiteman 2004).

Regarding saliva functionality, a significant increase in total protein concentration accompanied by a reduction in amylase activity was observed in the MeHg-exposed group, suggesting a qualitative impairment of salivary gland function rather than an enhancement of secretory performance. At the molecular level, this dissociation between total protein content and enzymatic activity may be explained by the high affinity of MeHg for sulfhydryl groups, which are essential for proper protein synthesis, folding, and enzymatic function (Farina et al. 2011; Nogueira et al. 2021). By binding to these groups, MeHg may selectively compromise the synthesis and structural integrity of functional proteins such as amylase, while simultaneously promoting the accumulation of non-enzymatic or stress-related proteins, thereby increasing total protein levels without preserving enzymatic activity.

In addition, experimental evidence also suggests that chronic MeHg exposure can induce structural alterations and atrophy of salivary glands, leading to dysfunctional secretion and leakage of intracellular proteins into saliva (Aragão et al. 2022; Nascimento et al. 2021a; Farias-Júnior et al. 2017; Bittencourt et al. 2017). Together with the disruption of redox balance, these mechanisms likely exacerbate protein instability and enzymatic inactivation, contributing to increased total protein concentration alongside reduced amylase activity.

In contrast, supplementation with clarified açaí mitigated these deleterious outcomes, as indicated by increased antioxidant capacity and reduced levels of pro-oxidant biomarkers, which may help preserve cellular integrity and redox-dependent enzymatic function, thereby attenuating the reduction in amylase activity and normalizing saliva composition. Given that salivary amylase plays a key role in carbohydrate digestion, bacterial bioadhesion, and innate immune defense, alterations in its activity may reflect underlying glandular dysfunction and contribute to impaired saliva quality, oral health disturbances, and reduced quality of life (Boehlke et al. 2015; Farias-Júnior et al. 2017; Müller et al. 2023; Alnaeem et al. 2025). Moreover, because its composition is modulated by physiological conditions and exposure to toxic agents, salivary amylase represents an efficient biomarker of systemic and local alterations (Nascimento et al. 2021b).

Although our study presents a preclinical experimental design, it successfully characterized the antioxidant properties in response to the toxicological challenge posed by Hg intoxication, particularly in relation to oxidative stress. Some limitations of our study, which justify further research, are the need to evaluate more specific pathways of MeHg damage by verifying the correlation with the protective properties of açaí. In addition, it is necessary to investigate the effects of chronic exposure on other salivary parameters and repercussions of oxidative stress on morphological aspects, which could serve as a link between the biochemical and functional mechanisms evaluated here.

## Conclusion

This study demonstrates that clarified açaí juice exerts a protective effect on the major salivary glands and saliva of rats exposed to MeHg. Beyond the experimental findings, these results have relevant public health implications for the Amazon region, where chronic Hg exposure remains a major concern due to environmental contamination and dietary habits. Given that açaí is a widely consumed, culturally relevant, and locally available food, its antioxidant properties may represent a complementary dietary strategy to mitigate Hg-induced biological damage in exposed populations. Nevertheless, further studies are required to determine the long-term safety, efficacy, and translational applicability of clarified açaí supplementation in the context of heavy metal exposure.

## Data Availability

All quantitative and qualitative data that served to support the results of this study are included in the article itself.
